# Scavenger receptor class B type I regulates cellular cholesterol metabolism and cell signaling associated with breast cancer development

**DOI:** 10.1186/bcr3483

**Published:** 2013-09-24

**Authors:** Christiane Danilo, Jorge L Gutierrez-Pajares, Maria Antonietta Mainieri, Isabelle Mercier, Michael P Lisanti, Philippe G Frank

**Affiliations:** 1 Department of Stem Cell Biology and Regenerative Medicine, Kimmel Cancer Center, Thomas Jefferson University, Philadelphia, PA 19107, USA; 2 Manchester Breast Centre & Breakthrough Breast Cancer Research Unit; Paterson Institute for Cancer Research; Institute of Cancer Sciences; Manchester Academic Health Science Centre, University of Manchester, Manchester, UK; 3 Department of Biochemistry and Molecular Biology, Kimmel Cancer Center, Thomas Jefferson University, Philadelphia, PA 19107, USA

## Abstract

**Introduction:**

Previous studies have identified cholesterol as an important regulator of breast cancer development. High-density lipoprotein (HDL) and its cellular receptor, the scavenger receptor class B type I (SR-BI) have both been implicated in the regulation of cellular cholesterol homeostasis, but their functions in cancer remain to be established.

**Methods:**

In the present study, we have examined the role of HDL and SR-BI in the regulation of cellular signaling pathways in breast cancer cell lines and in the development of tumor in a mouse xenograft model.

**Results:**

Our data show that HDL is capable of stimulating migration and can activate signal transduction pathways in the two human breast cancer cell lines, MDA-MB-231 and MCF7. Furthermore, we also show that knockdown of the HDL receptor, SR-BI, attenuates HDL-induced activation of the phosphatidylinositol 3-kinase (PI3K)/protein Kinase B (Akt) pathway in both cell lines. Additional investigations show that inhibition of the PI3K pathway, but not that of the mitogen-activated protein kinase (MAPK) pathway, could lead to a reduction in cellular proliferation in the absence of SR-BI. Importantly, whereas the knockdown of SR-BI led to decreased proliferation and migration *in vitro*, it also led to a significant reduction in tumor growth *in vivo*. Most important, we also show that pharmacological inhibition of SR-BI can attenuate signaling and lead to decreased cellular proliferation *in vitro*. Taken together, our data indicate that both cholesteryl ester entry via HDL-SR-BI and Akt signaling play an essential role in the regulation of cellular proliferation and migration, and, eventually, tumor growth.

**Conclusions:**

These results identify SR-BI as a potential target for the treatment of breast cancer.

## Introduction

Breast cancer is estimated to have affected >200,000 women in the United States in 2012 and will be responsible for approximately 40,000 deaths, making it the second leading cause of cancer deaths [1]. Recently, attention has focused on examining the role of components of the diet, such as cholesterol, in breast cancer development. Plasma cholesterol levels are often lower in patients with advanced lung [2] and breast cancers [3-5], a phenomenon known as the “preclinical effect of cancer” [6]. This effect is believed to be consequential and not causal [6] but does suggest a role for cholesterol in cancer. Several epidemiologic studies have shown a positive correlation between elevated high-density lipoprotein cholesterol (HDL-C) levels and breast cancer risk [5,7-9], and recent data have corroborated this claim [10-16]. *In vivo* works have suggested that hypercholesterolemia induced by diet and/or genetic background leads to increased tumor burden and metastasis in murine breast cancer models [10,12]. *In vitro* analyses have shown that human breast cancer cell lines exhibit increased proliferation and migration in the presence of HDL [11,13,15-17]. The effect of cholesterol on breast cancer may be attributed to several of its properties and functions. Cholesterol is the precursor of bioactive steroid hormones such as estrogen. It is also necessary for the formation of plasma membrane microdomains known as lipid rafts [18]. Lipid rafts are believed to organize signaling molecules in the plasma membrane and, as a result, have been implicated in the development of human cancers [19]. Therefore, cholesterol may play an essential role in the regulation of tumor growth [20,21].

The HDL lipoprotein is an important carrier of plasma cholesterol and can function as a signaling molecule by initiating MAPK and AKT signaling pathways and stimulate migration in endothelial cells [22-24]. The activation of these signaling pathways is dependent on HDL binding to the HDL receptor, the scavenger receptor class B, type I (SR-BI), and subsequent lipid transfer to the cell [25-27]. SR-BI functions as the HDL receptor and has been shown to mediate the selective transfer of cholesteryl ester from HDL molecules to cells in a process known as the selective HDL-cholesteryl ester uptake [28]. Its role in the development of atherosclerosis has been well documented [28], but its role in cancer has not been extensively investigated. Nevertheless, SR-BI has been implicated in prostate [29] and breast cancer [15,30]. In the case of breast cancer, SR-BI protein levels were found to be increased in malignant tissue samples compared with the normal surrounding tissue [30].

In the present study, we have examined the role of HDL and SR-BI in the regulation of cellular signaling pathways in breast cancer cell lines and in the development of tumors in a mouse xenograft model. Our data show that HDL can stimulate migration and can activate signal-transduction pathways in the two human breast cancer cell lines, MDA-MB-231 and MCF7. Furthermore, we also show that knockdown of the HDL receptor, SR-BI, attenuates HDL-induced activation of the MAPK and PI3K/Akt pathways in both cells lines. A more detailed analysis reveals that SR-BI regulates signaling pathways via Akt activation, and the regulation of SR-BI expression or activity can limit tumor development in a mouse model.

## Methods

### Materials

The following antibodies were used: SR-BI was from Novus Biologicals, Inc. (Littleton, CO, USA). CD31 antibody was from Abcam, Inc. (Cambridge, MA, USA). Phospho-Erk1/2 (T202/Y204), Erk1/2, Phospho-Akt (S473), and Akt were from Cell Signaling Technology, Inc. (Beverly, MA, USA). GAPDH was from Fitzgerald Industries International (Acton, MA, USA), and β-Actin was from Sigma-Aldrich Corp. (St. Louis, MO, USA). Anti-mouse secondary antibody was from Thermo Fisher Scientific, Inc. (Rockford, IL, USA), and anti-rabbit secondary antibody was from BD Biosciences (San Jose, CA, USA). The signaling inhibitors U0126 and LY294002 were from Cell Signaling Technology and Sigma-Aldrich, respectively. BLT-1 was from EMD Millipore (Billerica, MA, USA).

### Cell culture

MCF7 cells were obtained from the American Type Culture Collection (ATCC) (Manassas, VA, USA), and MDA-MB-231 cells were as previously described [31]. MDA-MB-231 and MCF7 cells were grown in Dulbecco modified Eagle media (DMEM) containing 10% fetal bovine serum (FBS) in an incubator kept at 37°C with 5% CO_2._

### Purification of lipoproteins

Human plasma was obtained from adult female volunteers. Approval for the use of human plasma was obtained from the Office of Human Research at Thomas Jefferson University, and consent was obtained from the volunteers for the use of their plasma samples. Lipoproteins (LDL (1.019-1.063 g/ml), HDL_2_ (1.063 to 1.125 g/ml), HDL_3_ (1.125 to 1.21 g/ml] were separated by density-gradient ultracentrifugation, as previously described [32]. HDL_3_ was dialyzed against PBS, 0.5 m*M* EDTA, 1% NaN_3_. In experiments with MCF7 cells, lipoproteins were stripped of estrogens with activated charcoal to remove endogenous steroid hormones, as previously described [16].

### Migration and invasion assays

Migration was assayed by using modified, noncoated Boyden chambers from BD Biosciences (San Jose, CA, USA). Both MDA-MB-231 and MCF7 cells were cultured in 10-cm dishes in DMEM containing 10% FBS. Cells were harvested, counted, and washed 3 times with PBS. Cells were resuspended in DMEM containing 1% BSA, and 5.0 × 10^4^ cells were added to the upper chambers. The bottom chambers contained 1% FBS or 100 μg/ml lipoprotein in 1% BSA. Cells were incubated at 37°C overnight. At the end of the experiment, upper chambers were swabbed with a cotton swab to remove nonmigrating cells, stained with crystal violet, and quantified by using phase-contrast microscopy. Migration was quantified by counting the number of cells in five separate fields at 10× magnification. Invasion assays were performed as described earlier for transwell-migration assays, but used Matrigel-coated Boyden chambers (BD Biosciences). The upper chambers contained cells in 1% BSA, and the bottom chambers contained 1% FBS or 100 μg/ml lipoprotein in 1% BSA.

### Immunoblot analysis

Cells were lysed in radioimmunoprecipitation assay (RIPA) buffer containing protease and phosphatase inhibitors from Roche Applied Science (Indianapolis, IN, USA) and Sigma-Aldrich Corp., respectively, and prepared and analyzed as previously described [33]. In brief, proteins were separated by sodium dodecylsulfate/polyacrylamide gel electrophoresis (SDS-PAGE) and transferred to a nitrocellulose membrane. Membranes were blocked in 5% BSA in Tris-buffered saline containing 0.1% Tween-20 (TBS-T). Primary antibodies were diluted in 5% BSA in TBS-T and incubated for either 1 hour at room temperature or overnight at 4°C. Membranes were washed 3 times in TBS-T, and incubated with horseradish peroxidase-conjugated secondary antibody for 1 hour at room temperature. Membranes were washed 3 times in TBS-T, and visualized by using Pierce Chemiluminescent Substrate from Thermo Fisher Scientific, Inc.

### Knockdown of SR-BI

MDA-MB-231 and MCF7 stably transfected cell lines were produced by lentiviral transduction. Lentiviral particles containing a pool of three short-hairpin RNA (shRNA) constructs targeted against SR-BI were purchased from Santa Cruz Biotechnology, Inc. (Santa Cruz, CA, USA). Control lentiviral particles containing scrambled shRNA sequences were also purchased from Santa Cruz. Cells were transduced with the lentiviral particles according to manufacturer’s protocol. Two days after transduction, target cells containing either control shRNA (shCTL) or shRNA against SR-BI (shSRBI) were selected by using 2.5 μg/ml puromycin. Successful knockdown was verified by immunoblot analysis.

### Cholesterol-content determination

Cells were grown to confluence in 10-cm dishes in the presence of complete media (DMEM, 10% FBS). In half of the plates, cholesterol was extracted with isopropanol. The extract was dried down in glass tubes under nitrogen and resolubilized in a smaller volume of isopropanol. Cholesterol content was determined by using the Cholesterol E kit from Wako Chemicals USA, Inc. (Richmond, VA, USA) as per the manufacturer’s instructions. In the other plates, cells were lysed with 0.5 NaOH, and lysates were collected. Protein concentration was determined with the bicinchoninic acid assay (BCA) from Thermo Fisher Scientific, as per the manufacturer’s instruction. Total cellular cholesterol levels were calculated by dividing the total cholesterol by the total protein per dish.

### ^3^H-Thymidine incorporation proliferation assays

Cell proliferation was measured with [^3^H]-thymidine incorporation to assess DNA synthesis and proliferation. MDA-MB-231 cells were seeded (5.0 × 10^4^ cells/well) in 12-well plates in 1 ml of DMEM containing 10% fetal bovine serum (FBS) and grown overnight. The following day, media was aspirated, cells were washed twice with PBS, and were serum-starved with 1% fatty acid free bovine serum albumin (BSA) for 1 hour. Media containing FBS, 1% BSA, or 1% BSA with lipoproteins (100 μg/ml) and 1 μCi/ml of ^3^H-thymidine from Perkin Elmer (Waltham, MA, USA) was added to cells. Cells were incubated for 6 hours, at which time media was removed, cells were washed twice with PBS and incubated in 10% trichloroacetic acid to precipitate DNA. Cells were solubilized in 0.1 *M* NaOH and 1% SDS. Radioactivity was measured by liquid scintillation counting. Protein concentration was determined by using the BCA assay.

### Tumor studies

All mice were housed and maintained in a barrier facility at the Kimmel Cancer Center at Thomas Jefferson University. Mice used in this study were athymic nude mice obtained from Taconic (Hudson, NY, USA). Animal protocols used for these studies were approved by the Institutional Animal Care and Use Committee of Thomas Jefferson University. MDA-MB-231 (10^6^) cells containing either shRNA targeted against SR-BI (shSRBI) or control shRNA containing scrambled shRNA (shCTL) were subcutaneously injected in the flanks of 7- to 9-week-old nude mice. MCF7 cells (5 × 10^6^) were orthotopically injected into the mammary fat pad of 9-week-old athymic nude mice implanted with slow-release 17β-estradiol pellets (0.36 mg/pellet, 60 days) from Innovative Research of America (Sarasota, FL, USA). Four weeks after injection, tumors were excised, weighed, and the volume was determined by using the formula (width^2^ × length)/2. Half of each tumor was flash frozen and stored at -80°C and subsequently homogenized and lysed in RIPA buffer for immunoblot analysis, as previously described [33]. The other half was fixed in formalin for 24 hours and then used to prepare paraffin-embedded sections.

### Immunohistochemical analysis

Paraffin-embedded tumor sections were deparaffinized in xylene and rehydrated. Antigen retrieval was performed in 10 m*M* citrate buffer pH6 for 10 minutes by using a pressure cooker. Endogenous peroxidase activity was blocked with 3% H_2_O_2_, and sections were blocked in 10% goat serum obtained from Vector Laboratories, Inc. (Burlingame, CA) and incubated with primary antibody overnight at 4°C. Sections were washed 3 times with PBS, incubated with biotinylated secondary antibody for 30 minutes, followed by HRP-conjugated streptavidin for 30 minutes by using a Streptavidin-HRP kit from Dako North America, Inc. (Carpinteria, CA, USA). After three washes in PBS, the presence of bound antibody was visualized by using 3,3′-diaminobenzidine (DAB). Slides were counterstained with hematoxylin, dehydrated, and mounted with coverslips.

### TUNEL assay

Apoptosis was measured with TUNEL assay by using the TUNEL-based ApopTag Peroxidase In Situ Apoptosis Detection Kit from Millipore (Temecula, CA, USA), as per manufacturer’s instructions. In brief, paraffin-embedded tumor sections were de-paraffinized and rehydrated. Sections were treated with 20 μg/ml proteinase K from Roche Applied Science (Indianapolis, IN, USA) for 15 min at room temperature and washed, and peroxidase activity was blocked by incubation in 3% hydrogen peroxide for 5 minutes. Sections were then incubated with equilibration buffer, followed by incubation in TdT enzyme for 1 hour at 37°C. After washing, sections were incubated with HRP-conjugated antibody directed again digoxigenin for 30 minutes at RT, washed, and apoptotic positive cells were visualized by using DAB. The percentage of apoptotic cells was quantified by dividing the number of TUNEL-positive cells by the total number of cells observed in four distinct fields per section.

### Statistical analyses

All values are expressed as the mean ± standard deviation (SD). The Prism 4.0 program (GraphPad Software, Inc., San Diego, CA, USA) was used for statistical analysis. Statistical significance was examined by using the Student’s *t* test or ANOVA when appropriate.

## Results

### HDL_3_ stimulates migration and activates Akt and Erk1/2 in MCF7 and MDA-MB-231 cells

**Figure 1:**
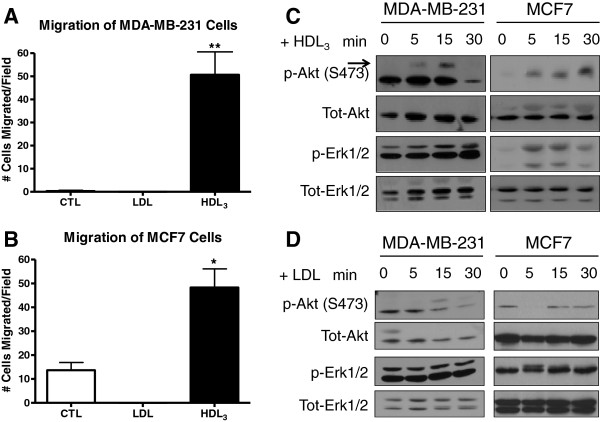
**HDL**_**3 **_**induces migration and acts as a signaling molecule in MDA-MB-231 and MCF7 cells. ** Migration of MCF7 **(A)** and MDA-MB-231 **(B)** cells was induced by HDL_3_. Migration of MCF7 and MDA-MB-231 cells was increased when HDL_3_, but not LDL, was used as a chemoattractant compared with CTL (1% BSA alone) in Boyden Chamber assays. Stained cells were quantified by using phase-contrast microscopy. Columns represent the mean number of migrated cells; bars represent ± standard deviation (SD) (*n* = 3). The results obtained with CTL and HDL_3_ groups are significantly different. No statistical difference was found between CTL and LDL groups. **(A)** *P < 0.05, Student's *t* test. **(B)** **P < 0.01, versus 1% BSA by Student's *t* test). Results shown are representative of three independent experiments. **(C)**. MDA-MB-231 and MCF7 cells were incubated for the indicated amounts of time with 100 μg/ml HDL_3._ Whole-cell lysates were analyzed by Western blotting for the indicated proteins. The arrow indicates the protein corresponding to phospho-Akt. Results are representative of three independent experiments. **(D)** MDA-MB-231 and MCF7 cells were incubated for the indicated amounts of time with 100 μg/ml LDL. Whole-cell lysates were analyzed by Western blotting for the indicated proteins. The arrow indicates the protein corresponding to phospho-Akt. Results are representative of three independent experiments.

Previous studies have shown that HDL can induce migration of endothelial cells [24]. In cancer, tumor cell migration represents the initial step associated with the development of metastasis [34]. To examine the effect of HDL on breast cancer cell migration, we studied the effect of lipoproteins on the migration of two breast cancer cell lines, MCF7 and MDA-MB-231. Interestingly, we found that when HDL_3_ was used as the chemoattractant, it induced migration of both MCF7 and MDA-MB-231 cells by 3.5- and 61-fold, respectively, compared with the controls (CTL) as a chemoattractant (Figure 1A, B). Interestingly, LDL had no effect on the migration of either MCF7 or MDA-MB-231 cells (Figure 1A, B). Because lipoproteins, specifically HDL, can act as signaling molecules in endothelial cells and prostate cancer cells and activate Akt and MAPK pathways [23,35], we examined their effect on signaling in MCF7 and MDA-MB-231 cells. However, HDL_3_ (100 μg/ml) stimulated the activation of Erk1/2 and Akt in both MCF7 and MDA-MB-231 cells (Figure 1C). A modest increase in the phosphorylation of Erk1/2 was observed in MDA-MB-231 cells after 30 minutes of incubation with HDL_3_. However, a more-robust and quicker response was observed in MCF7 cells (Figure 1C). Additionally, HDL_3_ rapidly activated Akt in both cell lines, an effect that was prolonged in MCF7 cells (Figure 1C).

These results indicate that HDL_3_ can function as a signaling molecule in these two breast cancer cell lines. LDL had a modest effect on Akt activation, and no effect on Erk1/2 activation in either MDA-MB-231 or MCF-7 cells was observed (Figure 1D).

### Knockdown of the HDL receptor, SR-BI, attenuates the effects of HDL_3_ on signaling in MDA-MB-231 and MCF7 cells

**Figure 2:**
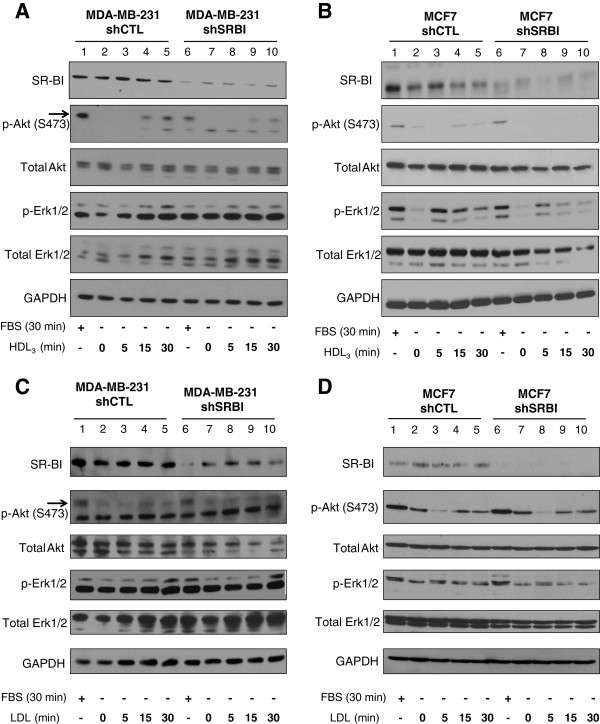
**Knockdown of SR-BI attenuates HDL**_**3**_**-induced signal transduction in MDA-MB-231 and MCF7 cells. (A**,**B)** MDA-MB-231 and MCF7 cells were stably transduced by lentiviruses carrying either shRNA against SR-BI (shSRBI) or control shRNA (shCTL). Cells were incubated with either 10% FBS for 30 minutes or with 100 μg/ml HDL_3_ for the indicated times. Whole-cell lysates were analyzed by Western blot for the indicated proteins. The arrow indicates the protein corresponding to phospho-Akt. GAPDH was used as loading control. **(C**,**D)** MDA-MB-231 and MCF7 cells were stably transduced by lentiviruses carrying either shRNA against SR-BI (shSRBI) or control shRNA (shCTL). Cells were incubated with either 10% FBS for 30 minutes or with 100 μg/ml LDL for the indicated times. Whole-cell lysates were analyzed by Western blot for the indicated proteins. The arrow indicates the protein corresponding to phospho-Akt. GAPDH was used as loading control. Note that, in panel **C**, the image was spliced (between lines 5 and 6) to eliminate an irrelevant line.

In the following experiments, we examined the effect of downregulating the HDL receptor, SR-BI, on signaling in MDA-MB-231 and MCF7 cells. As demonstrated in Figure 2, we were able to successfully downregulate SR-BI in both MDA-MB-231 cells (Figure 2A) and MCF7 cells (Figure 2B). Knockdown of SR-BI was achieved by stable transduction of a pool of lentiviral particles containing shRNA sequences specific for SR-BI (shSRBI). shCTL cells were created by stable transduction of lentiviral particles containing a scrambled version of the shRNA. Knockdown of SR-BI was assessed by Western blot analysis. In MDA-MB-231 cells, SR-BI expression was reduced by 5.3–fold, and in MCF7 cells, SR-BI expression was reduced by fourfold (Figure 2A).

To determine the role of SR-BI on the regulation of signaling pathways, both shCTL and shSRBI MDA-MB-231 and MCF7 cells were serum starved overnight and then incubated in media containing 10% FBS for 30 minutes or 100 μg/ml of HDL_3_ for 0, 5, 15, and 30 minutes, as indicated. We found that the activation of Akt was greatly reduced in the shSRBI cells compared with the shRNA control cells. Similar results were obtained with both MDA-MB-231 and MCF7 cell lines in the presence of FBS (lanes 1 and 6 in Figure 2A and B). Consistent with the results presented in Figure 1C, HDL_3_ was able to stimulate the activation of Akt in both cell lines in a time-dependent manner. However, activation of Akt in shSRBI MDA-MB-231 cells was greatly reduced when stimulated by HDL_3_ for 15 and 30 minutes (Figure 2A, lanes 9 and 10), compared with the Akt activation observed in shCTL MDA-MB-231 cells when stimulated by HDL_3_ for the same periods (Figure 2A, lanes 4 and 5). Similar results were obtained in MCF7 cells. In that case, Akt activation was reduced in the shSRBI MCF7 cells when stimulated by HDL_3_ for 15 and 30 minutes (Figure 2B, lanes 9 and 10), compared with shCTL MCF7 cells stimulated by HDL_3_ for the same periods (Figure 2B, lanes 4 and 5).

Finally, Erk1/2 appeared to be constitutively active in MDA-MB-231 cells (Figure 2A, lanes 1 to 10). However, almost no change in Erk1/2 activation was detected in shSRBI MDA-MB-231 cells treated with HDL_3_ for 30 minutes (Figure 2A, lane 10) compared with shCTL MDA-MB-231 treated with HDL_3_ for 30 minutes (Figure 2A, lane 5). This effect was in contrast with observations made with MCF7 cells (Figure 2B). In shCTL MCF7 cells, HDL_3_ rapidly stimulated Erk1/2 activation, reaching a peak at 5 minutes (Figure 2B, lane 3) but maintaining a sustained effect at 30 minutes (Figure 2B, lanes 4 and 5). Activation of Erk1/2 in shSRBI MCF7 cells followed a similar pattern, but the intensity of activation was greatly reduced (Figure 2B, lanes 7–10). These results suggest that downregulation of SR-BI in MDA-MB-231 and MCF7 cells attenuates signaling via the AKT and MAPK pathways. Additionally, our results show that the interaction between HDL and SR-BI regulates activation of these signaling pathways.

Finally, the effect of LDL was also tested in these cell lines. Results presented in Figure 2C and 2D demonstrate that the downregulation of SR-BI in MDA-MB231 and MCF7 cells had no effect on the regulation of Akt and Erk1/2 activation by LDL.

### Knockdown of the HDL receptor, SR-BI, inhibits proliferation and migration of MDA-MB-231 cells

**Figure 3:**
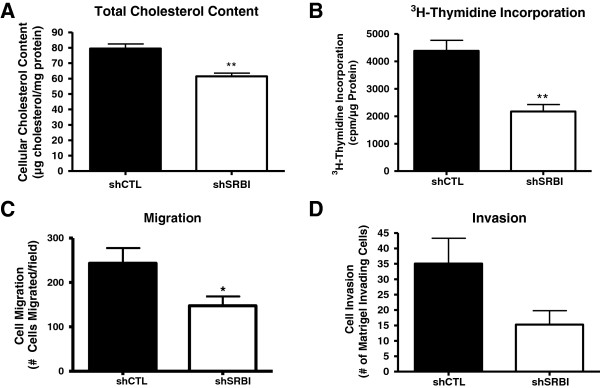
**Knockdown of the HDL receptor, SR-BI, inhibits proliferation, migration, and invasion of MDA-MB-231 cells. (A)** Cholesterol content of shCTL and shSRBI MDA-MB-231 cells. Cholesterol was extracted from cells with isopropanol, and total cholesterol was measured enzymatically by using a colorimetric assay. Protein concentration was also determined, and total cellular cholesterol content was expressed as the amount of cholesterol per milligram of protein. Columns represent the mean cholesterol content, and bars represent ± SD. shCTL MDA-MB-231 cells display significantly more cholesterol than shSRBI MDA-MB-231 cells (**P < 0.01 by Student's *t* test). **(B)** Knockdown of SR-BI inhibits proliferation of MDA-MB-231 cells. MDA-MB-231 cells containing control shRNA (shCTL) or shSRBI were incubated in the presence of ^3^H-thymidine for 6 hours in DMEM containing 10% FBS. Proliferation was measured by ^3^H-thymidine incorporation. Columns represent the mean ^3^H-thymidine incorporation (cpm/μg protein); bars represent ± (SD). The results obtained for shCTL and shSRBI cells are significantly different (*P < 0.01 by Student's *t* test). Results are representative of three independent experiments. **(C)** Knockdown of SR-BI reduces cellular migration. Cellular migration of shCTL and shSRBI MDA-MB-231 cells was assessed for 24 hours in a Boyden chamber by using 1% FBS as a chemoattractant. Columns represent the mean number of migrated cells from three independent experiments, and bars represent ± standard deviation (SD), (*n* = 12). The results obtained from the shCTL and shSRBI groups are significantly different (*P < 0.05 versus shCTL, as determined with Student's *t* test). **(D)** Knockdown of SR-BI leads to a marginally significant reduction in cellular invasion. Invasion of shCTL and shSRBI MDA-MB-231 cells was assessed by Matrigel-coated Boyden chamber assays by using 1% FBS as a chemoattractant. Columns represent the mean number of invaded cells from three separate experiments (*n* = 12); bars represent ± SD (*P* = 0.0517, Student's *t* test).

We observed decreased signaling in shSRBI MDA-MB-231 cells compared with shCTL MDA-MB-231 cells in the presence of FBS. Therefore, we used media containing 10% FBS for the remainder of the experiments described later. FBS contains large amounts of lipoproteins and provides suitable ligands for SR-BI. Previous studies have shown that expression of SR-BI in COS-7 cells significantly increased cholesterol mass in these cells compared with control vector-transfected cells [36]. To determine whether cellular cholesterol homeostasis was affected by the downregulation of SR-BI, we quantified the cholesterol content of shCTL and shSRBI MDA-MB-231 cells. Under basal conditions, shCTL MDA-MB-231 cells contained a significantly greater (1.3-fold increase) (*P <* 0.01) total cholesterol content compared with shSRBI MDA-MB-231 cells (Figure 3A).

Previous studies have shown that a mutant of SR-BI inhibits proliferation of the luminal B subtype of human breast cancer cells, MCF7, in the presence of HDL [30]. Further, to investigate the role of SR-BI in a triple-negative (lacking the estrogen receptor (ER), progesterone receptor (PR), and Her2) basal B subtype breast cancer cell line, we determined the effect of knocking down SR-BI on the proliferation of MDA-MB-231 cells. The proliferation of shSRBI MDA-MB-231 cell was reduced by twofold compared with the proliferation observed with shCTL MDA-MB-231 cells (*P* < 0.01) (Figure 3B). Knockdown of SR-BI also significantly reduced cellular migration (*P <* 0.05) by 1.65-fold (Figure 3C). Finally, a reduction of SR-BI protein levels was associated with a marginally significant reduction of the ability of MDA-MB-231 cells to invade (Figure 3D) (*P* = 0.0517).

### Pharmacologic inhibition of SR-BI reduces proliferation and signal transduction in MDA-MB-231 cells

**Figure 4:**
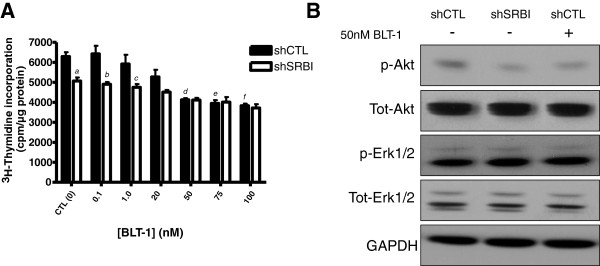
**Inhibition of SR-BI by the small-molecule BLT-1 inhibits proliferation of MDA-MB-231 cells. (A)** Pharmacologic inhibition of SR-BI in MDA-MB-231 cells inhibits cellular proliferation. MDA-MB-231 shCTL cells were incubated in the presence of the indicated doses of BLT-1 and ^3^H-thymidine added to the culture media for 6 hours. At this time, the assay was stopped, and lysates were collected. Columns represent the mean ^3^H-thymidine incorporation (cpm/μg protein); bars represent ± SD. Results obtained with shSRBI MDA-MB-231 CTL (0 n*M* BLT-1) are significantly different from those obtained with shCTL 0 n*M* BLT-1 (CTL) (*a*, *P <* 0.001), shCTL 0.1 n*M* BLT-1 (*b, P <* 0.001), and shCTL 1.0 nM (*c*, *P <*0.05). Results obtained with shCTL MDA-MB-231 CTL (0nM BLT-1) were significantly different from those obtained with shCTL 50 nM BLT-1 (*d*, *P <*0.001), shCTL 75 n*M* BLT-1 (*e*, *P <* 0.001), and shCTL 100 n*M* (*f*, *P <* 0.001). Significance was determined by one-way ANOVA with Tukey's post-test analysis. **(B)** Pharmacologic inhibition of SR-BI attenuates signaling in MDA-MB-231 cells. Western blot analysis was performed with extracts obtained from shSRBI MDA-MB-231 cells treated with vehicle, shCTL MDA-MB-231 cells treated with vehicle, and shCTL MDA-MB-231 cells treated with BLT-1 (50 n*M*). Cells were serum starved overnight, incubated with vehicle alone or BLT-1 for 3 hours, and then treated with 10% FBS for 30 minutes.

To elucidate the inhibitory effect of SR-BI ablation on proliferation and signal transduction obtained by molecular biologic means, we treated cells with the pharmacologic inhibitor of SR-BI, BLT-1 [37]. Previous work identified BLT-1 as a specific inhibitor of SR-BI function. BLT-1 has been shown to act by blocking the transfer of lipids from HDL particles to cells [37]. The IC_50_ for this compound was determined to be 50 n*M*[37]. The ability of BLT-1 to regulate proliferation was evaluated in the presence of varying concentrations of this inhibitor. Accordingly, BLT-1 could inhibit growth of shCTL MDA-MB-231 cells in a dose-dependent manner (Figure 4A). At doses >50 n*M*, BLT-1 treatment could significantly decrease proliferation of shCTL MDA-MB-231 cells compared with the control untreated cells (*P <* 0.001 for concentrations of 50 n*M*, 75 n*M*, and 100 n*M* compared with shCTL MDA-MB-231 incubated with 0 n*M* BLT-1). Importantly, there was no significant effect of BLT-1 treatment on the proliferation of shSRBI MDA-MB-231 cells: Proliferation of shSRBI cells treated with concentrations of BLT-1 between 0.1 n*M* and 100 n*M* was not statistically different from that of vehicle-treated shSRBI cells. A statistical difference between untreated shSRBI MDA-MB-231 cells and shSRBI MDA-MB-231 cells treated with 100 nM BLT-1 was also detected. This observation may be due to the presence of some residual SR-BI protein. Interestingly, no significant difference was found between shCTL MDA-MB-231 cells treated with BLT-1 at doses >20 n*M* and vehicle-treated (0 n*M*) shSRBI MDA-MB-231 cells. Taken together, these results suggest that downregulation or pharmacologic inhibition of SR-BI has similar effects on MDA-MB-231 proliferation.

We also examined the effect of BLT-1 on signal transduction in these cells. In agreement with the finding described in Figure 2A, Akt activation in shSRBI MDA-MB-231 cells treated with FBS for 30 minutes was reduced compared with shCTL MDA-MB-231 cells (Figure 4B). Similar results were obtained with shCTL MDA-MB-231 cells with treated BLT-1. Akt activation was reduced in the treated shCTL MDA-MB-231 cells compared with untreated control cells. Finally, SR-BI knockdown or pharmacologic inhibition had no effect on Erk1/2 activation (Figure 4B) compared with the control cells. Collectively, these data suggest that Akt activation may be mediated, in part, by SR-BI, and the downregulation of SR-BI is responsible for the observed reduction in the cellular proliferation.

### Inhibition of PI3K, not MEK1/2, inhibits growth of shCTL MDA-MB-231 cells

**Figure 5:**
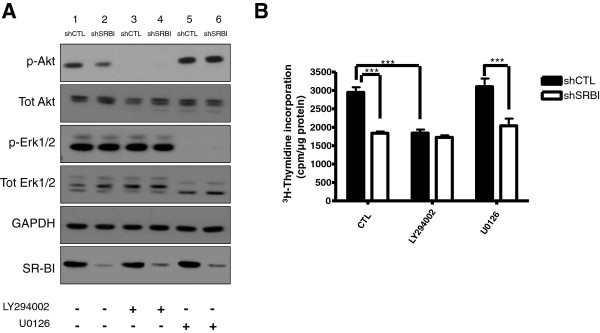
**Inhibition of PI3K, not MEK1/2, prevents proliferation of MDA-MB-231 cells. (A)** LY294002 and U0126 effectively inhibit Akt and Erk1/2 activation in MDA-MB-231 cells. Serum-starved shCTL and shSRBI MDA-MB-231 cells were incubated with or without the inhibitors LY294002 (15 μ*M*) or U0126 (10 μ*M*) for 2 hours. Medium containing 10% FBS was added for 30 minutes, cells were lysed, and whole-cell lysates were analyzed by Western blot for the indicated proteins. GAPDH was used as a loading control. **(B)** PI3K inhibition reduces cellular proliferation of shCTL MDA-MB-231 cells. shCTL and shSRBI MDA-MB-231 cells were incubated with culture media containing either LY294002 (15 μ*M*) or U0126 (10 μ*M*) and ^3^H-thymidine for 6 hours, at which time, the assay was stopped, and lysates were collected. Columns represent the mean [^3^H]thymidine incorporation (cpm/μg protein); bars represent ± SD. Results obtained from vehicle-treated (CTL) shCTL MDA-MB-231 cells are significantly different from those obtained with shSRBI MDA-MB-231 cells (**P < 0.001, one-way ANOVA with Tukey's post-test analysis). Results obtained from vehicle-treated shCTL MDA-MB-231 cells (CTL) are significantly different from those obtained with shCTL MDA-MB-231 cells treated with LY294002. Note that proliferation of shCTL MDA-MB-231 cells treated with U0126 was not significantly different from that observed with vehicle-treated shCTL MDA-MB-231 cells. Proliferation of shSRBI MDA-MB-231 cells treated with LY294002 or U0126 was also not significantly different from that observed with vehicle-treated shCTL MDA-MB-231 cells. Results are representative of three independent experiments.

To elucidate the mechanism by which SR-BI knockdown inhibits proliferation, we used pharmacologic agents to inhibit PI3K and MAPK signaling pathways. We show that the PI3K inhibitor, LY294002, abolished FBS-induced activation of Akt (Figure 5A) in shCTL and shSRBI MDA-MB-231 cells. Importantly, PI3K inhibition significantly reduced proliferation of shCTL MDA-MB-231 cells to levels similar to those observed with untreated shSRBI MDA-MB-231 cells (Figure 5B). In addition, PI3K inhibition had no effect on the proliferation of shSRBI MDA-MB-231 cells, suggesting that downregulation of SR-BI in these cells was sufficient to inhibit proliferation. Conversely, U0126-induced inhibition of MEK1/2, which activates Erk1/2, did not affect proliferation of shCTL MDA-MB-231 or shSRBI MDA-MB-231 cells (Figure 5B). Erk1/2 activation, however, was significantly reduced by inhibition of MEK1/2 in both cell types (Figure 5A). These results suggest that the MAPK pathway does not play a significant role in SR-BI-mediated signaling and proliferation, unlike the PI3K pathway.

### Knockdown of SR-BI results in decreases in *in vivo* tumor growth of MDA-MB-231 and MCF7 cells

**Figure 6:**
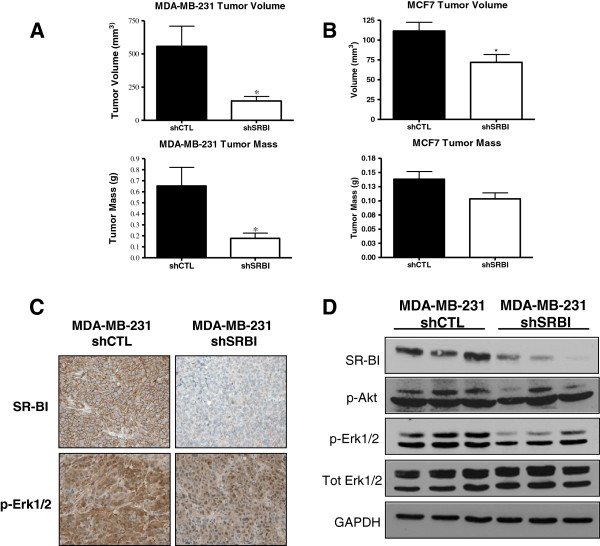
**Knockdown of SR-BI inhibits xenograft tumor growth *in vivo. *(A)** Knockdown of SR-BI in MDA-MB-231 cells decreases tumor growth *in vivo*. Athymic nude mice were injected with 10^6^ MDA-MB-231 cells carrying control shRNA (shCTL) or shSRBI. Four weeks after injection, mice were killed, and tumors were excised. Tumors were weighed and volume determined by using the formula (length × width^2^)/2. Columns represent the mean volume and mass, respectively; bars represent ±SD. Tumors obtained with shCTL cells were significantly larger by weight and volume than were the tumors obtained with shSRBI. (*P < 0.05; *n* = 14 shCTL, *n* = 13 shSRBI) **(B)** Knockdown of SR-BI in MCF7 cells decreases tumor growth *in vivo.* MCF7 cells (5 × 10^6^) were orthotopically injected into the mammary fat pads of 9-week-old athymic nude mice implanted with slow-release 17β-estradiol pellets (0.36 mg/pellet, 60 days). Four weeks after injection, mice were killed, and the tumors were excised. Tumors were weighed and volume determined by using the formula (length × width^2^)/2. Columns represent the mean volume and mass, respectively; bars represent ± SD. The shCTL tumors were significantly larger by volume than were the shSRBI tumors (*P < 0.05; *n* = 8 shCTL, *n* = 7 shSRBI). **(C)** shCTL tumors display increased expression of proliferative markers compared with shSRBI tumors. Immunohistochemistry was performed to determine expression patterns of SR-BI and pErk1/2 in MDA-MB231 tumors. **(D)** The shCTL tumors display increased expression of proliferative markers compared with shSRBI tumors. Three tumors per cell type were analyzed by Western blot analysis for the indicated proteins. GAPDH was used as a loading control.

To assess the effect of SR-BI knockdown *in vivo*, we subcutaneously injected shSRBI and shCTL MDA-MB-231 cells into the flanks of nude mice. Four weeks after injection, tumors were excised from dead mice, and mass and volume were measured. Tumors obtained with shCTL MDA-MB-231 were significantly larger than those obtained from shSRBI MDA-MB-231: tumor volume and mass were increased by 3.8-fold and 3.7-fold, respectively (Figure 6A). To determine the role of SR-BI in tumor growth in MCF7 cells, shCTL and shSRBI MCF7 cells (5 × 10^6^) were orthotopically injected into the mammary fat pad of athymic nude mice after implantation with slow-release 17β-estradiol pellets. Four weeks after injection, mice were killed, tumors were excised, and mass and volume of tumors were determined (Figure 6B). Tumors obtained with shCTL MCF7 were 1.5-fold larger than those obtained with shSRBI MCF7 (*P <* 0.05), and 1.3-fold larger by mass (marginally significant).

To elucidate the mechanism by which SR-BI regulates tumor formation, tissue immunohistochemical analyses (Figure 6C) and immunoblot analyses of homogenized tumors (Figure 6D) were performed. Immunohistochemistry analyses demonstrated the reduction in SR-BI protein expression in shSRBI MDA-MB-231-derived tumors compared with shCTL MDA-MB231. Results also revealed that levels of the proliferative marker, pErk1/2, were decreased in shSRBI MDA-MB-231 tumors, compared with those observed in control tumors. Consistent with *in vitro* findings, pAkt levels were decreased in shSRBI MDA-MB-231 tumors compared with those observed in control tumors.

**Figure 7:**
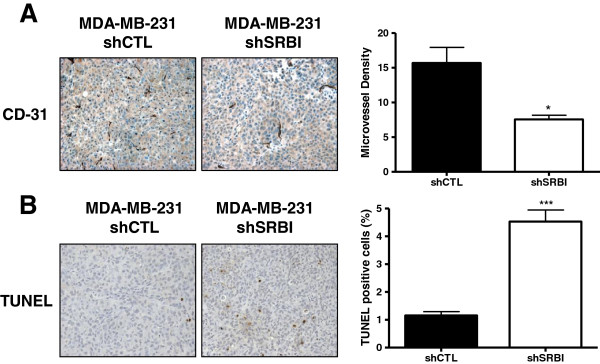
**Knockdown of SR-BI inhibits xenograft tumor growth *in vivo. *(A)** Knockdown of SR-BI results in decreased angiogenesis in MDA-MB-231 xenograft tumors. Immunohistochemistry was performed to determine expression of CD31. CD31 staining was quantified by averaging the number of vessels per field in five separate fields per sample. Columns represent the mean microvessel density; bars represent ± SD. Microvessel density was statistically greater in shCTL MDA-MB-231 tumors compared with shSRBI MDA-MB-231 cells (*P < 0.05 Student's *t* test; *n* = 4 per group). **(B)** Knockdown of SR-BI results in increased apoptosis in MDA-MB-231 xenograft tumors. Images shown are representative of TUNEL assay staining. Percentage of TUNEL-positive cells was calculated by dividing the number of TUNEL-positive cells by the total number of cells in four separate fields. Columns represent the percentage of TUNEL-positive cells; bars represent ± SD. TUNEL staining is statistically increased in shSRBI MDA-MB-231 tumors compared with shCTL MDA-MB-231 tumors (***P < 0.001, Student's *t* test, shCTL *n* = 3, and shSRBI *n* = 4).

Because cholesterol has been shown to play a role in the regulation of angiogenesis [38], microvessel density in the tumors was assessed by staining tumor sections for CD31, a specific marker of endothelial cells (Figure 7A). A significant increase in microvessel density was observed with tumors obtained from shCTL MDA-MB-231 cells compared with those obtained from shSRBI MDA-MB-231 cells. These data suggest that SR-BI can regulate angiogenesis in these tumors.

Finally, SR-BI has been shown to activate Akt [23], which may inhibit apoptosis, thereby promoting cell survival [39]. Therefore, we assessed apoptosis with TUNEL staining in tissue sections obtained from shCTL and shSRBI MDA-MB-231 xenograft tumors (Figure 7B). As anticipated, we observed a significant increase in apoptosis in shSRBI MDA-MB-231 tumors compared with shCTL MDA-MB-231 tumors.

## Discussion

In the present study, we examined the role of HDL and its receptor, SR-BI, in breast cancer development and progression. We found that HDL_3_ stimulates migration and activates signaling pathways such as MAPK and PI3K in two breast cancer cell lines. Inhibiting selective HDL-cholesteryl ester uptake by knocking down or pharmacologically inhibiting SR-BI resulted in an attenuation of cell-signaling events induced by HDL. Additionally, loss of SR-BI resulted in decreased proliferation, migration, and tumor growth of MDA-MB-231 cells. These findings suggest that regulating cholesterol metabolism and cellular signaling pathways via SR-BI may be linked and may additionally identify new targets associated with tumor progression.

### HDL, signal transduction, and cellular migration

HDL has a well-established role in the etiology of atherosclerosis, particularly in reverse cholesterol transport, whereby HDL removes excess cholesterol molecules from peripheral tissues and returns them to the liver for excretion or recycling [40]. In addition, HDL functions in a number of other cellular processes, including inhibition of apoptosis in macrophages [41], induction of migration in endothelial cells [24], and the initiation of cell-signaling events in multiple cell types [22,23,42]. Although clinical studies have suggested that plasma HDL levels may be correlated with increased breast cancer risk [5,7-9,12], the mechanisms by which HDL exerts its effect have yet to be elucidated. HDL has been shown to activate Erk1/2 in fibroblasts [23,42], Chinese hamster ovary cells [22], endothelial cells [23], and prostate cancer cells [35,43]. Studies have also shown that it can activate Akt in endothelial [23] and prostate cancer cells [35,43]. Interestingly, the activation of Erk1/2 [44] and Akt [39,45] has been implicated in several human cancers, including breast cancer.

In the present study, we established a role for HDL as a mediator of signal transduction in two breast cancer cell lines. Consistent with the results obtained in other cell types, we found that, in both MCF7 and MDA-MB-231 cells, incubation with HDL_3_ induces a rapid activation of both Erk1/2 and Akt signaling pathways. These novel findings in breast cancer suggest that HDL may regulate various signaling pathways and may therefore alter tumor progression.

In the present study, we found that HDL can induce migration of two breast cancer cell lines, MCF7 and MDA-MB-231, suggesting that HDL may play a role in the early stages of metastasis. This finding is consistent with previous studies showing that HDL can stimulate migration of endothelial cells [24,46]. Interestingly, the observed migration was shown to be mediated by SR-BI [24]. By contrast, a recent study reported that HDL inhibits migration of MDA-MB-231 in Boyden chamber assays [14]. However, in this study, the investigators used serum as the chemoattractant, and HDL was added to the upper chamber, thereby measuring the ability of HDL to prevent cellular migration induced by serum. By contrast, our method allows the analysis of the role of HDL in the regulation of cellular migration and therefore allows a direct measurement of the capability of HDL to induce migration of MDA-MB-231 cells. As a result, our results indicate that HDL may play a role in the pathogenesis of breast cancer, especially in the later stages.

### SR-BI, signal-transduction regulation, and tumor formation

SR-BI has been implicated as a mediator of several cell-signaling events in the context of atherosclerosis [22,23,25-27]. Previous studies have shown that HDL binding to SR-BI and subsequent lipid transfer are sufficient to activate Src, which subsequently activates the PI3K/Akt and MAPK pathways [25,27]. In endothelial cells, one of the downstream effectors of Akt is eNOS, which catalyzes the production of NO. The results obtained in the present study are consistent with the hypothesis that SR-BI may also play a role in signal transduction in the context of cancer. In agreement with this hypothesis, upon knockdown or pharmacologic inhibition of SR-BI in MDA-MB-231 cells, Akt activation was significantly reduced, suggesting that SR-BI may be mediating this response. In addition, downregulation of SR-BI was accompanied by a reduction of total cholesterol levels in MDA-MB-231 cells. These results are consistent with reports that indicate that the cholesterol flux mediated by SR-BI plays a role in the regulation of signal-transduction initiation [26]. In our model, decreased total cholesterol levels may represent a reduction in SR-BI-mediated cholesterol flux and therefore significantly reduce signal-transduction activation. SR-BI also binds LDL, which can, like HDL, promote the cellular entry of cholesteryl ester. Although LDL, may promote the entry of cholesteryl ester via SR-BI, it is not sufficient to induce migration of breast cancer cells, and it does not appear to alter Akt activation (Figure 2C, D). Taken together, our data suggest that both cholesteryl ester entry via HDL-SR-BI and Akt activation are required for cellular proliferation and migration, and, eventually, tumor growth.

Activation of the PI3K/Akt pathway promotes growth, survival, and proliferation [45] and has been implicated in a variety of human cancers [39]. Importantly, Akt is aberrantly hyperactivated in approximately 40% of breast cancers [39]. We observed a reduction in proliferation and migration in the SR-BI-knockdown cells compared with control cells in association with reduced Akt activation. These results suggest that SR-BI may mediate the activation of Akt and its downstream effects in the presence of HDL. Mechanistically, we showed that the inhibition of the PI3K/Akt pathway results in significantly reduced proliferation of shCTL MDA-MB-231 cells, similar to the reduction in proliferation observed in shSRBI MDA-MB-231 cells. Importantly, no further reduction in proliferation of shSRBI MDA-MB-231 cells was detected upon inhibition of the PI3K/Akt pathway. Taken together, these data suggest that reduced Akt activation observed in the shSRBI MDA-MB-231 cells may be responsible for reduced proliferation of these cells compared with shCTL MDA-MB-231 cells.

Previous studies suggested a role for SR-BI in the etiology of breast cancer. Cao *et al*. [30] showed that expression of SR-BI is increased in human breast tumors compared with the normal surrounding tissue. They also demonstrated that recombinant expression of a mutant form of SR-BI, which lacked the carboxyl-terminal tail of the protein, could inhibit proliferation of breast cancer cells. Their study further suggested that this effect was possibly due to reduced Akt activation. Our study is the first to demonstrate directly that Akt activation is reduced when SR-BI is knocked down or pharmacologically inhibited. Furthermore, in agreement with the previously mentioned study [30], we showed that proliferation of MDA-MB-231 cells was significantly inhibited by downregulation of SR-BI protein levels and by pharmacologic inhibition of SR-BI. In addition, we observed that SR-BI knockdown inhibits migration. This finding may suggest a role for SR-BI in the initiation of metastasis. Finally, we demonstrated that knockdown of SR-BI in MDA-MB-231 cells can lead to reduced tumor growth *in vivo* accompanied by increased activation of Erk1/2 and Akt, and an increase in cellular apoptosis. In MCF7 cells, knockdown of SR-BI also led to reduced xenograft tumor growth.

Previous studies have shown that Akt can inhibit apoptosis through a variety of mechanisms, including the phosphorylation of BAD, thus preventing cytochrome *c* release from mitochondria and the direct inhibition of the caspase activation cascade [39]. Increased cellular cholesterol levels have been shown to increase Akt activation and decrease apoptosis in prostate cancer cells [47]. Consistent with these observations, a recent study showed that inhibition of xenograft tumor growth could be achieved with colon cancer cells that re-express the ATP-binding cassette transporter A1 (ABCA1) [48]. ABCA1 is a lipid transporter that mediates the efflux of cellular cholesterol to lipid-free apolipoprotein A-I [49]. Furthermore, in this study, re-expression of ABCA1 resulted in decreased mitochondrial cholesterol content and increased release of cytochrome *c*, which ultimately led to increased apoptosis. Our work also indicated that SR-BI knockdown can significantly reduce apoptosis in xenograft tumors, as shown by TUNEL staining. Consequently, a reduction in the levels of cellular cholesterol content may be responsible, at least in part, for the decreased apoptosis observed in our model. Taken together, these data also suggest an important role for cholesterol in the regulation of cellular signaling pathways and tumor formation. Importantly, excess cellular cholesterol accumulates in the form of esterified cholesterol. Previous works and ours suggest that the accumulation of esterified cholesterol may lead to a modification of signaling pathways associated with proliferation and migration in tumors. Consistent with this hypothesis, increasing cellular esterified cholesterol levels have been shown to induce cellular proliferation and enhance invasiveness of tumor cell lines [50]. Conversely, the inhibition of cholesterol esterification has been shown to have the reverse effect [51,52].

## Conclusions

In summary, our results suggest that HDL and SR-BI have pro-oncogenic activity and can induce migration and activate signal-transduction pathways responsible for cellular proliferation and tumor formation in two breast cancer cell lines. Additionally, knockdown or pharmacologic inhibition of SR-BI could attenuate signaling mediated by HDL and inhibit proliferation, migration, and tumor growth. Taken together, these findings identify SR-BI and HDL as potential therapeutic targets for the treatment of breast cancer.

## Abbreviations

ABCA1: ATP-binding cassette A1; Akt: Protein kinase B; BLT1: Blocks lipid transport-1; BSA: Bovine serum albumin; eNOS: Endothelial nitric oxide synthase; ER: Estrogen receptor; Erk: Extracellular-signaling-related kinase; FBS: Fetal bovine serum; HDL: High-density lipoprotein; HDL-C: High-density lipoprotein-cholesterol; LDL: Low-density lipoprotein; PI3K: Phosphatidylinositol 3-kinase; SR-BI: Scavenger receptor class B, type I.

## Competing interests

The authors declare that they have no competing interests.

## Authors’ contributions

PGF supervised all aspects of this research and the preparation of this manuscript. CD, MPL, IM, and PGF participated in the design of the study. CD, JLGP, IM, and MAM carried out the experimental data acquisition. CD, JLGP, MPL, MAM, and PGF performed data analyses. All authors read, critically revised, and approved the final manuscript.
